# Non-Sterile Gloves as a Source of Radiation-Tolerant Microorganisms

**DOI:** 10.3390/microorganisms11122859

**Published:** 2023-11-25

**Authors:** Celine Cabeau, Romain Bolle-Reddat, James Hauschild, Gerald McDonnell

**Affiliations:** 1 Microbiological Quality & Sterility Assurance, DePuy Synthes, Chemin Blanc 38, 2400 Le Locle, Switzerland; cidini@its.jnj.com (C.C.); rbollere@its.jnj.com (R.B.-R.); 2 Microbiological Quality & Sterility Assurance, Johnson & Johnson, Building 930 East, 1000 Route 202 South, Raritan, NJ 08869, USA; jhausch@its.jnj.com

**Keywords:** radiation, sterilization, resistance, water quality, microbial contamination

## Abstract

Radiation methods are widely used for disinfection and sterilization applications. Microorganisms demonstrate known, variable tolerance levels to inactivation with lower doses of ionizing and non-ionizing radiation based on multiple mechanisms of resistance in their structures and nucleic acid repair mechanisms. The radiation dose required to ensure microbial inactivation during sterilization is typically based on the understanding and routine monitoring of the natural population and resistance of microorganisms on products exposed to radiation sterilization processes. This report describes the isolation of *Roseomonas mucosa* in a device manufacturing environment that was detected during routine device bioburden and dose verification monitoring. Sources of Gram-negative bacteria in the environment were investigated. Non-sterile examination gloves used during manufacturing were found to be a persistent source of *R. mucosa* and other microbial contaminants. The source of contamination was determined to be from the glove manufacturing process. Maintenance and routine microbiological controls during glove manufacturing, including water systems, are required to reduce the risks of gloves being a source of unexpected microbiological contamination.

## 1. Introduction

Non-ionizing and ionizing radiation are widely used methods of disinfection and sterilization. Radiation sources, depending on their levels of associated energy, can lead to the disruption of the structure and function of the various macromolecules that make up microbiological life [1,2]. The lethal effects are due to multiple ionization events and particularly the disruption of the structure and function of nucleic acids such as DNA. Higher energy, ionizing radiation methods such as gamma radiation, electron beam and X-ray radiation are used as sterilization methods due to their reliable and broad-spectrum antimicrobial activity against microorganisms. These methods and associated validation criteria are used internationally under the requirements of ISO 11137 series of standards [3,4].

Despite the broad-spectrum antimicrobial effects of such energy sources, microorganisms are naturally known to display different levels of intrinsic and sensitivity tolerance or resistance to inactivation [5]. This is expected due to their various structures and known mechanisms of resistance [2]. For example, non-enveloped viruses and dormant forms of microorganisms (e.g., bacterial and fungal spores) demonstrate higher resistance in comparison to enveloped viruses and vegetative bacteria or fungi due to their structures. These differences and resistance mechanisms are well-described in the literature for all types of disinfection and sterilization methods [2]. Of particular note, certain types of vegetative bacteria have been demonstrated to have the ability to unexpectedly survive higher doses of radiation due to naturally occurring and cumulative methods of tolerance. These species may remain sensitive to radiation at typical user dose ranges used for medical device sterilization applications but are reported to have higher tolerance levels to radiation doses that rapidly inactivate other vegetative bacteria. Radiation-tolerant bacteria include *Roseomonas* and *Deinococcus* species and remain the subject of detailed investigation [6,7]. The mechanisms of resistance have been studied in these bacteria, and the major mechanisms described are found to preserve, protect, or even repair the microbial DNA against damage during exposure, as the nucleic acid is the main target of radiation antimicrobial activity. These mechanisms include the presence of multiple copies of the bacterial genome, efficient DNA repair mechanisms, and other factors such as the presence of carotenoid pigments. The production of pigments often presents as brightly colored colonies of these bacteria on agar culturing, often as strongly colored reds and pinks [2]. The pigments act as free-radical scavengers and can protect the cell from damage due to hydroxyl radicals, which are formed on contact with radiation and contribute to the overall antimicrobial effects in vegetative cells [8]. These and other resistance mechanisms can also show cross-resistance to other antimicrobial methods, such as the impact of oxidizing agents [2]. 

A particular example is *R. mucosa*, a slow-growing, Gram-negative, strictly aerobic, typically pink-pigmented coccobacilli. The species name derives from the Latin term *mucosa* (mucous or slime), referring to the mucoid bacterial colonies when cultured on selective agar media [9,10]. They are often isolated from water, including pure water sources. The ability of these and other bacteria to survive in such systems is important to consider as their persistence due to their ability to adapt to antimicrobial methods can lead to cross-contamination risks on the use of the water during various manufacturing processes. Periodic monitoring for the presence and control of these types of bacteria in critical manufacturing environments (such as for medical devices) is best practice particularly for products that are to be sterilized by radiation methods. It is good manufacturing practice to isolate and remediate the source of the bacteria in these systems—for radiation sterilization applications, if this is not considered, then the presence of higher populations of bacteria with increased resistance levels to radiation will require higher dose levels of radiation to ensure a consistent and complaint process.

Radiation is one of the main sterilization modalities used internationally. Sterilization validation methods follow applicable standards [3,4] and commonly utilize a bioburden or bioburden-overkill approach. These are based on the knowledge of the quantity and resistance of viable organisms present on the product to be sterilized, known as the bioburden [11]. This determination and ongoing monitoring allow for the appropriate minimal dose to be defined during validation and routine verification to ensure product sterility. The bioburden evaluation is used to estimate the radiation dose to inactivate that population on a typical product, which is verified by testing the sterility of representative products on exposure to that minimum dose [12]. This is based on prior knowledge from the routine monitoring of devices sterilized by radiation; for example, when conducted over a 10-year period, it has been shown that spore-forming and non-spore-forming bacteria can be, although rarely, responsible for such dose verification failures across a wide range of products [13,14]. 

However, exposure to the minimum verification dose also confirms that higher doses used for routine radiation sterilization of the products provide a robust process and specifically a defined Sterility Assurance Level (SAL) in accordance with the standard and regulatory requirements. The reduced radiation (or ‘verification’) dose is periodically applied to normal manufactured product samples and verified for sterility by culturing [12]. In case of test failure under these conditions, routine products manufactured in the interim are evaluated to ensure that the required SAL under normal (higher) dose ranges has been maintained. 

We report here the investigation of the periodic isolation of *R. mucosa* in product bioburden and pre-sterilization over an extended period with no obvious source identified from routine environmental sampling. Although these levels were low, they were eventually linked as the sources of periodic, failed product radiation dose verification screening results. The subsequent investigation of the novel source of the contaminants is described, leading to the conclusion that the unsuspected source is non-sterile examination gloves used during manufacturing. The detection of significant microbial contamination on examination gloves may be a further concern in other applications or uses of the gloves in industrial and medical applications. 

## 2. Materials and Methods

### 2.1. Product Bioburden Determination

The device master product under investigation was representative of a series of sterile-labeled medical devices used in sports medicine surgery and manufactured under similar environmental conditions. The products were assembled using components from different controlled sources that were primarily packaged under cleanroom conditions (Class 8 cleanroom; [15]) and then subsequently sterilized by a validated gamma radiation process. The product bioburden was routinely determined according to the method validated according to ISO 11737-1 ([11] described below). The bioburden test method was validated using the artificial inoculum method (using spores of *Bacillus atrophaeus* ATCC 9372 as the test inoculum), and the method demonstrated an extraction efficiency of ~57%. During an established bioburden monitoring program, 10 product samples were periodically taken from the manufacturing environment following final packaging in a cleanroom environment, and the device bioburden was estimated individually. Test devices were immersed in 450 mL of peptone buffer (3.5 g/L disodium hydrogen phosphate, 10 g/L peptone, 1.5 g/L potassium dihydrogen phosphate, and 5 g/L sodium chloride) with 0.1% (*w*/*v*) Tween 80 and mechanically shaken for 10 min at room temperature (16–22 °C). The extracts were membrane-filtered through 0.45 mm filters and incubated on Tryptic Soy Agar (TSA; Sigma Aldrich, Darmstadt, Germany) for 3 days at 30–35 °C. The total product bioburden was determined by direct plate counting and applying the correct factor determined in the method validation (based on an extraction efficiency of 57%).

### 2.2. Gamma Radiation Exposure

Radiation sterilization validation was determined using a bioburden method based on VDmax [3,4]. The products were validated for and routinely sterilized with a range of 25–40 kGy. Based on periodic monitoring, the average bioburden level was shown to be ~15 colony forming units (CFU)/device and, in accordance with the standard, a verification dose of 7.6 kGy was determined to be applicable for test purposes to verify a SAL of 10^−6^ at a minimum, routine sterilization dose of 25 kGy. Ten product samples in a batch were periodically taken from the manufacturing environment following final packaging and subjected to the verification dose (7.6 kGy of g radiation, as confirmed by dosimetry in compliance with the standards; 3, 4). The samples were then aseptically unpacked, individually immersed into 1600 mL of Tryptic Soy Broth (TSB; Sigma Aldrich, Darmstadt, Germany) and incubated at 28–32 °C for 14 days. The presence or absence of growth was visually inspected and recorded. Any sample showing growth was subcultured by streaking onto TSA plates and incubated at 30–35 °C for 3–5 days for further analysis. Growth was inspected for purity, and colonies were selected for microbiological identification.

### 2.3. Microbiological Identification

Isolates were initially identified by Gram-staining and microscopic examination. Sub-cultured bacteria were identified by Matrix-Assisted Laser Desorption/Ionization Time-Of-Flight Mass Spectrometry (MALDI TOF MS) method using either the microflex^®^ LRF (Bruker; Billerica, MA, USA) or Biolog GENIII systems (Biolog; Hayward, CA, USA) to genus and species level using the commercial systems-associated databases.

### 2.4. Environmental Sampling

Routine environmental sampling at the manufacturing site (e.g., critical water, cleanroom surface and room air or compressed air monitoring) for microbial contamination was well-established at the manufacturing site. Analysis of these results over multiple years did not detect any potential significant source for *Roseomonas* prior to this detailed investigation. More detailed testing of other suspected sources was investigated at the site. These included non-critical water (e.g., used for handwashing) and various other manufacturing surfaces such as in entry/exit areas, cleanroom garments and accessory equipment/materials used in the clean room during manufacturing. 

For the sampling of water sources, 50 mL water samples were taken aseptically from three city water taps located in the entrance area of the cleanroom used for handwashing prior to entry. Each source was tested in duplicate. Samples were filtered onto individual filter membranes, transferred onto TSA and R2A agar (Sigma Aldrich, Darmstadt, Germany) and incubated at 30–35 °C for 3–5 days. Colony isolates, especially at the species level, including those presenting pigment formation (such as pinkish, red, or a pale mucous aspect), were examined by Gram staining. All Gram-negative bacteria were further identified by using MALDI-TOF MS. 

A total of 16 glove dispensers located either in the entrance area of the cleanroom or the cleanroom itself were tested for surface contamination using 55 mm TSA contact plates with neutralizer. Each plate was incubated at 30–35 °C for 4 days. As above, colonies were further examined by Gram staining and speciated by the MALDI-TOF MS method. 

Five replicates of pre-sterile gowns worn by cleanroom operators were randomly and aseptically selected following use in the cleanroom. Each gown was immersed in 1200 mL peptone buffer with 0.1% (*m*/*v*) Tween 80 and mechanically shaken for 10 min at room temperature conditions (16–22 °C). For each solution, an extract of 120 mL was membrane-filtered through 0.45 µm filters and incubated on TSA media for 7 days at 30–35 °C. The total product bioburden was determined by direct plate counting. Any growth was Gram-stained, and any Gram-negative bacteria were identified by the MALDI TOF MS method.

### 2.5. Determination of Glove Contamination

Initial environmental sampling, described above, suggests that examination (nitrile medical grade non-sterile) gloves used in the cleanroom during product packaging may be a potential and periodic source for *Roseomonas*. To enhance the detection method, multiple gloves of each designated sample lot were examined for bioburden, and the culturing incubation method was extended from 3 to 5 days. Initially, 20 gloves were sampled together from each of five different batches and from five different glove sizes directly from the manufacturer. Each 20-glove batch sample was eluted with 750 mL peptone buffer with 0.1% (*m*/*v*) Tween 80 by mechanically shaking for a minimum 10 min. Extracts were filtered onto individual filter membranes, transferred onto TSA agar plates and incubated at 30–35 °C for 5 days. Any growth was inspected for purity, and colonies were selected for subculturing and microbiological identification by the MALDI-TOF MS method. An additional series of tests was conducted by extracting 10 gloves of each designated lot/size with 400 mL peptone buffer with 0.1% (*m*/*v*) Tween 80 in sterile stomacher bags (Seward Stomacher 3500; VWR, Dietikon, Switzerland). Samples were loaded into a Seward Stomacher 400 Lab System (VWR, Dietikon, Switzerland), ensuring the glove samples were submerged and processed for 120 s at normal speed. Duplicate 150 mL rinsate aliquots from each sample bag were filtered onto individual filter membranes, transferred onto duplicate TSA agar and incubated at 30–35 °C for 5 days. 

## 3. Results and Discussion

**Figure 1:**
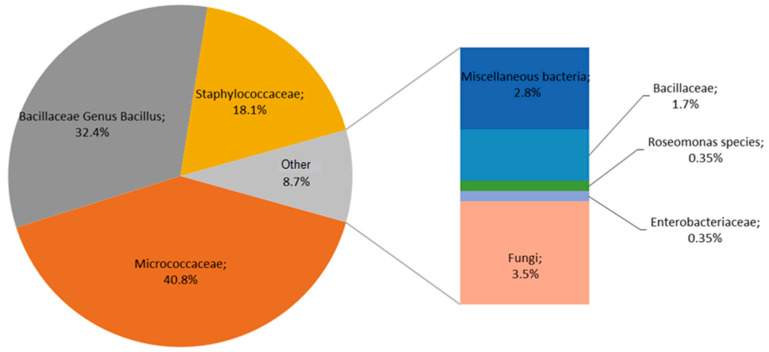
Qualitative summary of product bioburden conducted quarterly for the product family under investigation 2014–2017. Recovery frequency is shown as percentages.

**Figure 2:**
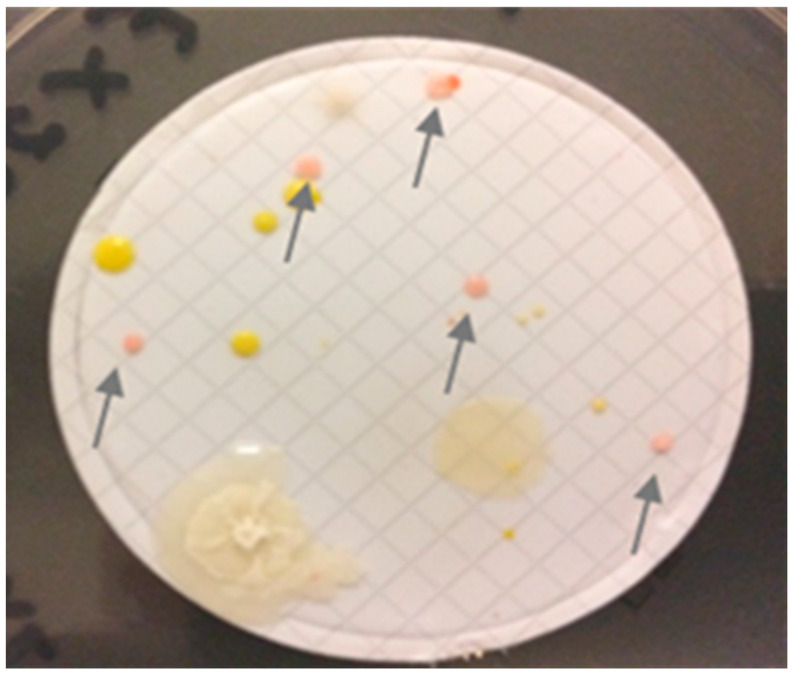
An example of the results of bioburden testing during the investigation, with examples of pigmented colonies (identified as *Roseomonas*) indicated with arrows.

Bioburden and dose verification testing were performed routinely for 60 product families representing all products packaged under aseptic (cleanroom) conditions at the manufacturing site prior to terminal radiation sterilization. The history of bioburden levels at the manufacturing site from 2014 to 2017 for all product families was consistently <100 CFU/device. Specific trend analysis at the time of this investigation demonstrated consistent bioburden levels of, on average, ~15 CFU/device for the product families under investigation, and this level was being used at this time to specify the verification dose requirements for the validated sterilization process. Qualitative analysis of the bioburden demonstrated typical environmental microorganisms for the manufacturing conditions described, consisting of 33% Micrococcaceae, 27% Bacillaceae (*Bacillus* sp.), 21% Staphylococcaceae, and others at 19%, generally and periodically represented by Gram-negative bacteria and fungi (Figure 1). *Roseomonas* sp. had been periodically identified at low concentrations and at <1% of product samples during the previous years (Figure 2). Investigations for any potential sources of *Roseomonas*, particularly on the investigation of any water sources at the manufacturing site, did not identify any particular source of contamination. However, it was also noted during the investigation that from 2014–2016, a total of two dose verification samples (an individual sample from each of two test batches of product) had failed testing and that both were from the same production line and test product family. The radiation sterilization standard requirements (for the VDmax method) allow for at least one out of 10 tests to demonstrate growth at the fractional radiation dose tested to provide an estimated SAL of 10^−1^ that can be extrapolated on a SAL of 10^−6^ at the higher, routine sterilization dose of 25–40 kGy [3,4]. 

In 2017, one gamma-radiation sterilization family also failed a routine, quarterly fractional dose audit verification sterility test, with 3 out of 10 samples demonstrating growth. This was considered significant and was further investigated. Sub-culturing of the failed dose audit samples identified *R. mucosa* (in two samples) and *Bacillus odysseyi* (from one sample). No obvious laboratory or irradiation process errors were detected. Subsequent repeat testing of this product family in compliance with the radiation standards successfully demonstrated passing results (exceeded the recommended dose augmentation requirements; 1, 3, 4). In this case, it is important to remember that the dose verification exposures in accordance with the standards are only designed to expose the product bioburden to a fraction of the dose normally used as an overkill process for routine sterilization (in this case, at a minimum of 25 kGy).

Despite the passing results in compliance with the standards, the product family and associated manufacturing conditions were subjected to more detailed investigations to understand potential sources and to reduce the risks of further occurrence. Historical bioburden data for the product family under investigation showed the presence of *Roseomonas* at a low percentage (0.35%) since 2014 (Figure 1). Previous attempts to identify potential sources were unsuccessful, including routine and additional environmental monitoring (including air, surface and water sampling). However, *Roseomonas* spp. were periodically recovered from a range of representative product bioburden families at the manufacturing site, suggesting a common source across those product families associated with cleanroom practices. Two common elements were identified on closer inspection of the different product manufacturing lines: the use of non-sterile nitrile gloves and the use of water for handwashing prior to glove donning and entry into the cleanroom. Both were subjected to further investigation. Microbiological testing of water used for handwashing at various sites prior to cleanroom entry did not show the presence of *Roseomonas*. However, the results did show pink pigmented colonies at one site that, on sub-culturing, was identified as a *Methylobacterium* sp. This risk was overall considered low as cleanroom operators performed hand hygiene prior to entering the cleanroom and were required to were gloves on product handling within the cleanroom. 

Particular attention was paid to the further investigation of examination gloves utilized within the cleanroom. Initial sampling of individual gloves samples periodically demonstrated low levels of bacteria; therefore, the sampling size was increased by pooling 10 gloves in triplicate testing to increase the sensitivity of detection. These results demonstrated on initial testing an average of 2 CFU/10 gloves of *R. mucosa* and 3 CFU/10 gloves of *Moraxella osloensis*. Further sampling identified that the levels of *R. mucosa* and types of other microorganisms varied depending on the various glove batches and sizes used in the cleanroom. The most frequently identified microorganisms in each of 5 sizes of gloves from a single manufacturer used in the cleanroom are summarized in Table 1. Only two sizes (XS and M) consistently demonstrated the presence of the *R. mucosa* strain during the investigation. The gloves were purchased in large, sealed bags and were found to be naturally contaminated in situ from unused, unopened bags. *R. mucosa* was not identified from other environmental sources in the cleanroom; however, the gloves were routinely dispensed into cleanroom-specific glove-dispensing boxes and environmental swabbing results from these boxes in one case identified the presence *M. osloensis*, demonstrating a potential link to cross-contamination from the gloves. Further testing was conducted on a larger sampling of the glove-dispensing boxes and, in addition, on several pre-sterilized overalls following normal use in the cleanroom. Testing was performed using TSA media contact plates by direct contact with the test surfaces, followed by incubation. Results from this analysis showed the presence of a single colony of *R. gilardii* from the glove-dispensing boxes and one colony of *R. mucosa* from one used gown, also suggesting a low level of cross-contamination from the glove source. 

*Roseomonas* sp., including *R. mucosa*, has been reported as being associated with patient infections. They are opportunistic pathogens and have been associated with bacteremia in otherwise immunocompromised patients [10]. Some outbreaks have been reported with *R. mucosa* linked with hospital environmental sources (particularly water [16]) and most often associated with the patient’s own skin flora [17]. Like many Gram-negative bacteria, they can often demonstrate resistance to certain types of antibiotics (e.g., β-lactams) and the ability to develop biofilms [18]. Interestingly, some commensal strains have been used for the treatment of atopic dermatitis, an inflammatory skin disease in animal and human studies [19].

In this investigation, *R. mucosa* and other strains of bacteria/fungi were isolated from fresh, unopened batches of examination gloves. Direct contact with gloves on product handling pre-sterilization was the cause of the detection of *Roseomonas* in routine bioburden and dose verification studies. Cross-contamination within the cleanroom was likely because of the detection of *Roseomonas* from environmental testing on glove dispenser boxes or gowns used in the cleanroom. These levels remained low in the environment overall, suggesting that the routine environmental cleaning-disinfection practices were sufficient to reduce cross-contamination rates from the general cleanroom environment. However, the risk of product contamination in a cleanroom is certainly from direct handling of products and product contact surfaces, therefore highlighting the risk of contaminated gloves. In this investigation, the bioburden levels and presence of bacteria with greater tolerance to radiation did not present a significant risk as the products are terminally sterilized in their primary packaging using a significantly higher dose of radiation during normal product manufacturing. The tolerant strains only survived at lower (dose verification) radiation doses. In addition, the resistance of the strains detected was not unusual and was within the known, expected resistance levels of bacteria to radiation and the requirements of the radiation sterilization standards [5]. *Roseomonas* was only detected on test devices at radiation doses at a fraction of the normal sterilization process in this case, but for other sterilization processes that may use lower sterilization doses, this may present a higher risk.

The use of clean but non-sterile gloves is common for the handling of products and materials in cleanrooms and, indeed, many clinical and other environments. Sterile gloves, in bulk or individually wrapped, are more commonly used in more critical environments, such as during aseptic manufacturing (e.g., in cleanrooms of class 5 or lower) or during the handling of patients during medical care. The cost and environmental impact of the use of sterile gloves is not warranted in low-risk applications, such as in the handling of non-sterile devices in cleanroom applications. At the same time, non-sterile gloves should not be a significant source of microbial contaminants, and the gloves investigated in this report demonstrated a range of microorganisms that may be linked to manufacturing and general environmental sources (Table 1). While some of these at low levels, such as sourced from the skin (e.g., staphylococci and micrococci) and the general environment (e.g., spore-forming bacilli) may be acceptable, the presence of other contaminants, such as higher levels of fungi and Gram-negative bacteria is more concerning. The presence of fungi may indicate a bigger concern from cleanroom or packaging concerns during glove manufacturing, as well as Gram-negative bacteria due to the potential development of biofilms in water systems used for the extrusion and rinsing of gloves during processing. The presence of radiation-tolerant bacteria, in our case, was an unexpected but manageable risk at the manufacturing site and ranged over time. However, it is important to consider that these organisms were identified on dried gloves over an extended time since manufacturing, which suggests that even higher concentrations of such contaminants may be present at the manufacturing source. The control of such contaminant sources at the manufacturing site is important to consider. 

An initial investigation of the typical sources of *Roseomonas* and other bacteria during the glove manufacturing process was initiated. Nitrile and other plastic gloves are typically produced by a process of dipping, chlorination and final water rinsing followed by drying and packaging. Although low levels of typical airborne bacteria would be expected under such controlled manufacturing environments, the levels of controls (e.g., cleanroom requirements for drying and packaging) and maintenance requirements of water used for rinsing were found to range dramatically depending on the glove manufacturer. It is likely, but we were unable to verify with the specific manufacturer that the source of variable levels of *R. mucosa* was due to inadequate maintenance and sanitization/disinfection of water rinsing systems during glove manufacturing. It is not practical or necessary in a wide range of applications to ensure that examination gloves are provided sterile, but it is recommended that microbiological quality controls are in place at glove manufacturing facilities to ensure that the gloves do not become a source of contamination. Glove suppliers should define appropriate glove specifications and perform risk assessments to identify critical process steps, adequate equipment specifications and monitoring programs in place for critical utilities such as water and compressed air. 

Good manufacturing principles are important in considering the potential risk of the development of radiation-tolerant microorganisms in manufacturing environments. In our case, the manufacturing and terminal sterilization of products using a radiation process was found to be robust enough to overcome the associated contamination risk due to the standard validation requirements [3,4]. Built into this validation approach is the known intrinsic resistance of microorganisms to radiation, and this has been well studied [2,5,13,14]. Intrinsic resistance is the basis for the standard distribution of resistances (SDR) defined in the radiation sterilization standards [3,4,20]. The SDR, used in validation methods 1 and VDmax described in the standard, is based on previous and detailed testing of radiation resistance of heterogeneous microbial populations [3,5]. The use of the SDR represents a maximal challenge to radiation sterilization, and the applicable verification dose to set a 10^−6^ SAL sterilization dose involves performing tests of sterility on product samples that have received doses of radiation significantly lower than the sterilization dose. The verification dose is therefore used to demonstrate that the radiation resistance of the product-associated bioburden is less than or equal to the radiation resistance of the microbial population representing the SDR, thereby verifying the efficacy of the actual 10^−6^ SAL sterilization dose. 

Similar to other physical and chemical disinfection and sterilization methods, spore-forming bacteria (and fungi) demonstrate some of the highest levels of natural resistance due to their structures. These include spore forms of *Bacillus*, *Paenibacillus*, *Clostridium*, *Aureobasidium* and *Brevibacillus* species [1,2]. However, the SDR also considered many vegetative (non-spore) bacteria that demonstrate an accumulation of intrinsic mechanisms of resistance that show similar, if not higher, levels of resistance to radiation doses than some bacterial spores [2]. These include vegetative cells of *Deinococcus*, *Methylobacterium* and *Roseomonas* species. As expected, their growth characteristics often present as highly pigmented colonies such as pinks, reds and coral over extended incubation times on growth media, but not in all cases, highlighting that pigmentation is only one of multiple mechanisms of resistance to radiation processes [21]. Similar to *Roseomonas*, *Methylobacterium* species are a cause of healthcare-associated infection, including infections in immunocompromised hosts. The ability of *Methylobacterium* species to form biofilms and to develop resistance to high temperatures, drying and disinfecting agents may explain the colonization of hospital environments and associated devices, e.g., endoscopes. They often demonstrate greater expression of tolerance to drying and biocides during the stationary phase of growth or under stressful situations as important survival mechanisms. Due to their cited slower growth rates, they can be easily missed during routine microbiological surveillance in clinical practices, such as in the monitoring of flexible endoscopes following processing [21]. In our case, *R. mucosa* was readily detected within the culture conditions utilized over 3 days of incubation, but the colonies were small, and we used a longer incubation time of 5 days to improve detection (Figure 2). In addition to pigmentation, the colonies also appeared to be mucoid, which is typical for these species. The mechanisms of radiation resistance are multi-factorial [2], and the pigments act as free-radical scavengers and can protect the cell from damage due to hydroxyl radicals generated during the radiation process [22]. Interestingly, during our investigation, the levels of pigment formation ranged considerably from pinks to browns; therefore, pigmentation alone may not be the best indication of radiation resistance in bacteria and in those cases where it may contribute, these effects may be subtle (depending on the radiation dose) and variable (depending on the growth phase and environmental conditions of the bacteria).

Overall, this report highlights the importance of detailed monitoring and excursion investigations of microbiological contamination levels in manufacturing environments. It is necessary to understand not only the quantity of detectable microorganisms but also their qualitative types (e.g., bacteria, fungi or speciation), as this can be important in assessing the risk to manufacturing processes. In our case, the risk was low to the manufacturing process, as the products were subsequently subjected to terminal sterilization in their primary packaging, and the potential for overgrowth of bacteria isolated was unlikely as the associated manufacturing process was not associated with water or sources of water. However, the investigation identified that non-sterile examination gloves can be a source of medical device bioburden levels and a potentially greater risk to other manufacturing or clinical situations. This is because they can have direct contact with the device surfaces during manufacturing, particularly when the manufacturing processes require a lot of manual handling during assembly processes. Although the use of sterile gloves can be considered in higher-risk situations, we would recommend that non-sterile gloves are practical for use when best practices in microbiological quality are considered at the glove manufacturing sites. We expect from our investigation that water systems (including for extrusion or rinsing) during glove manufacturing are at a high risk of Gram-negative bacterial contamination and biofilm development, thereby compromising the expected microbiological quality of the gloves. Although many types of bacteria will be inactivated or become unviable on drying gloves and the storage of gloves over time, certain types of bacteria, such as *Roseomonas* species can better tolerate drying conditions due to mechanisms of resistance natural in these bacteria. It is possible, under certain manufacturing conditions, that these will compromise product quality and, when applicable, pose a significantly higher burden to achieve terminal sterilization when utilizing radiation methods. 

## 4. Conclusions

Water systems used in manufacturing and other situations can be a source of Gram-negative bacteria due to biofilm development and persistence over time. Such systems, when not correctly designed and maintained, may be a particular source of microorganisms with a higher tolerance to antimicrobial processes. This is typically due to the presence of a stressed environment to bacteria that can allow for the survival and growth of certain types of Gram-negative bacteria. The stresses in the typical designs of pure water systems include chemical purity (thereby being poor in available nutrients), contact with low doses of radiation (due to the use of UV lights within the circulation system to control the potential presence of bacteria), the periodic use of oxidizing agents (for routine disinfection or sanitization of the water system) and finally, any drying systems following the use of the water on a surface. There are potential cross-resistance mechanisms to all of these stressed conditions that can allow for the persistence of radiation-tolerant microorganisms when these water systems are not maintained and monitored effectively [23]. Examples of these cross-resistance mechanisms include activation of SOS responses [22], efficient repair systems [24], protein structure and protective mechanisms [25], presence and overproduction of carotenoid pigments (acting as antioxidants; [8]), build-up of intracellular concentrations of manganese (as an antioxidant; [26]), cell wall structure [27] and higher G:C content giving greater stability to DNA molecules [27]. Overall, such environments may allow for the survival and promotion of bacteria with these naturally existing tolerance mechanisms; therefore, the correct design and routine monitoring of such systems in critical manufacturing applications is prudent. Non-sterile gloves may be an unexpected source of microbial contamination due to inadequate microbial controls during their manufacturing process and despite being provided dry for use due to these associated tolerant mechanisms.

## Figures and Tables

**Table 1:** Types of microorganisms demonstrated on the non-sterile gloves investigated.

Glove Size	Types of Microorganisms
Extra Small (XS)	Bacillaceae (*Bacillus* sp.) Staphylococcaceae Micrococcaceae Fungi Pseudomonaceae *R.mucosa*
Small (S)	Bacillaceae (*Bacillus* sp.) Fungi
Medium (M)	Bacillaceae (*Bacillus* sp.) Micrococcaceae Pseudomonaceae *R.mucosa*
Large (L)	Bacillaceae (*Bacillus* sp.) Fungi
Extra Large (XL)	Bacillaceae (*Bacillus* sp.) Fungi Micrococcaceae

## Data Availability

The data underlying this article will be shared upon reasonable request to the corresponding author.
